# Three-Dimensional Solution for the Vibration Analysis of Functionally Graded Rectangular Plate with/without Cutouts Subject to General Boundary Conditions

**DOI:** 10.3390/ma14227088

**Published:** 2021-11-22

**Authors:** Wenhao Huang, Kai Xue, Qiuhong Li

**Affiliations:** College of Mechanical and Electrical Engineering, Harbin Engineering University, Harbin 150001, China; huangwenhao@hrbeu.edu.cn (W.H.); xuekai@hrbeu.edu.cn (K.X.)

**Keywords:** three-dimensional vibration, FGMs plate, plate with circular cutouts, three-dimensional elasticity theory, Rayleigh–Ritz method, region mapping

## Abstract

Functionally graded materials (FGMs) structures are increasingly used in engineering due to their superior mechanical and material properties, and the FGMs plate with cutouts is a common structural form, but research on the vibration characteristics of FGMs plate with cutouts is relatively limited. In this paper, the three-dimensional exact solution for the vibration analysis of FGMs rectangular plate with circular cutouts subjected to general boundary conditions is presented based on the three-dimensional elasticity theory. The displacement field functions are expressed as standard cosine Fourier series plus auxiliary cosine series terms satisfying the boundary conditions in the global coordinate system. The plate with circular cutout is discretized into four curve quadrilateral sub-domains using the *p*-version method, and then the blending function method is applied to map the closed quadrilateral region to the computational space. The characteristic equation is obtained based on the Lagrangian energy principle and Rayleigh–Ritz method. The efficiency and reliability of proposed method are verified by comparing the present results with those available in the literature and FEM methods. Finally, a parametric study is investigated including the cutout sizes, the cutout positions, and the cutout numbers from the free vibration characteristic analysis and the harmonic analysis. The results can serve as benchmark data for other research on the vibration of FGMs plates with cutouts.

## 1. Introduction

The laminated composites are widely used in various engineering applications—such as aerospace, mechanical, civil, and automotive engineering—due to high specific strength and stiffness, light weight, and good thermal stability. However, stress-induced failures may occur through large in-plane stress, interlayer slip or transverse normal stress [1,2]. In order to overcome the adverse effects of laminated composites in mechanical properties, the engineering application of functionally graded materials (FGMs) was first proposed in 1984 by a group of aerospace scientists, due to a need for a type of material that can withstand high temperature difference in a space plane project [3]. The FGMs are a new type of advanced composite materials which are generally formed by two materials with smooth and continuous variation in specific direction from one surface to another, thus eliminating inter-laminar problems. The FGMs have received major attention since FGMs can effectively overcome these problems of traditional laminated composites. The corresponding specific material properties are obtained by the gradual variation in material properties and structure over volume fraction. The FGMs are designed to meet varying functionalities, and the FGMs plates and shells are the major structures owing to the wide variety of applications involved. Therefore, the study of the vibration characteristic analysis of FGMs plates and shells is an extremely important subject for engineers in structural design.

With the wide application of FGMs structures, a large amount of research work has been done on the vibration of FGMs plate and shell structures. The FGMs plate and shell structures are three-dimensional elastic bodies, the most accurate and effective way to solve the vibration problem is the three-dimensional elasticity theory; however, the three-dimensional elastic analysis of the structure leads to a partial differential equation system with three independent spatial variables, and the equation is often a transcendental equation about the frequency parameter. Thus, it is difficult to solve the transcendental equation, which requires the use of numerical root-finding algorithm. However, the difficulty of the numerical root-finding algorithm is how to determine the initial trial value, in addition, the algorithms cannot satisfy real-time requirements because of high computational cost. As a substitute, some appropriate assumptions were made about the displacement of the structure in the thickness direction, and the three-dimensional problems were simplified into two-dimensional ones with sufficient accuracy. These theories mainly include classical plate theory (CPT), first-order shear deformation theory (FSDT), and high-order shear deformation theory (HSDT). A brief review of these theories in the context of the FGMs structures will be presented in the following.

Chi and Chung [4,5] investigated three types of elastic, rectangular, and simply supported FGMs plates of medium thickness subject to transverse loading based on the classical plate theory and Fourier series. The results showed that only the bending stiffness formulations of FGMs plates are not similar with homogeneous plates due to their more complicated combination of material properties. Abrate [6] treated the FGMs plates as homogeneous plates, and selected a proper reference surface, the decoupling condition of the motion equation was derived to eliminate the coupling effect between the in-plane and bending deformations. Zhang and Zhou [7] used the physical neutral surface to study the theoretical analysis of the FGMs thin plates based on the classical plate theory. The stretching–bending coupling effect was eliminated, thus it is easier and simpler than classical laminated plate theory based on geometric middle surface.

Zhao et al. [8] presented the element-free-Ritz method for the free vibration analysis of FGMs plates. The first-order shear deformation plate theory was employed to account for the transverse shear strain and rotary inertia, and mesh-free kernel particle functions were used to approximate the two-dimensional displacement field. Hossenini-Hashemi et al. [9] presented analytical solutions for the free vibration analysis of FGMs plates, and a new formula for the shear correction factors used in Mindlin plate theory was obtained. In addition, using the Reissner–Mindlin plate theory, an exact closed-form procedure was presented by Hossenini-Hashemi et al. [10]. By introducing some new potential and auxiliary functions, the displacement fields were analytical obtained for FGMs plates configuration. Qu et al. [11] investigated a general formulation which was derived by means of a modified variational principle in conjunction with a multi-segment partitioning procedure on the basis of the first-order shear deformation shell theory for free, steady-state and transient vibration analysis of FGMs shells of revolution subjected to arbitrary boundary conditions. Ferreira et al. [12] used the global collocation method, the first-order and the third-order shear deformation plate theories, the Mori–Tanaka technique to homogenize material properties, and approximated the trial solution with multiquadric radial basis functions to analyze the free vibration of FGMs plates. Thai et al. [13] presented a simple first-order shear deformation theory which containing only four unknowns for solving the bending and free vibration analysis of FGMs plates by dividing the transverse displacement into bending and shear parts. Fallah et al. [14] used the extended Kantorovich method together with infinite power series solution to obtain semi-analytical solution for the governing equations of moderately thick rectangular FGMs plates.

Neves et al. [15] dealt with the free vibration problems of FGMs shells by radial basis functions collocation, according to a higher-order shear deformation theory that accounted for through-the-thickness deformation. Isogeometric analysis (IGA) [16,17] is an effective method to investigate the static and dynamic behavior of functionally graded carbon nanotube-reinforced composite plates. Phung-Van et al. proposed the higher-order shear deformation theory model and isogeometric analysis method based on Non-Uniform Rational B-Spline (NURBS) basis functions. Thanh et al. presented a size-dependent model based on the modified couple stress theory (MCST) and isogeometric analysis. Dozio [18] used the two-dimensional higher-order kinematic theories based on a powerful indicial notation and the state-space approach to solve the free vibration analysis of thick FGMs plates. Jodaei et al. [19] used the artificial neural network (ANN) method and the state-space-based differential quadrature method (SSDQM) to study the free vibration of FGMs annular plates. The results showed that the ANN method is a useful method to predict natural frequency, and the SSDQM has fast convergence speed.

Nie and Zhong [20] proposed a semi-analytical approach which used the state-space-method and one-dimensional differential quadrature method (SSM-DQM) to investigate the three-dimensional free and forced vibration analysis of FGMs circular plates. Malekzadeh [21] presented an accurate solution procedure based on the three-dimensional elasticity theory and the differential quadrature (DQ) method for the free vibration analysis of thick FGMs plates on two parameter elastic foundations. Dong [22] extended the Chebyshev–Ritz method proposed by Zhou [23,24] to the three-dimensional free vibration of FGMs annular plates. Huang et al. [25] employed the three-dimensional elasticity theory and a variational Ritz method to solve the vibration of rectangular parallelepipeds of FGMs with side cracks. Jin et al. [26] developed a unified and accurate solution method to deal with the free vibration of arbitrarily thick FGMs plates based on the linear, small-strain three-dimensional elasticity theory. The same method was performed by Zhao et al. [27] for the vibration analysis of thick functionally graded porous rectangular plates, and three kinds of porosity distributions including even, uneven, and the logarithmic-uneven were performed.

Plates or shells with cutouts are extensively used in engineering structures, cutouts are made to optimize structures, reduce the weight, or provide operational access to other parts of the structures. Comparatively speaking, most of the earlier investigations on plates with cutouts have been confined to isotropic plates, not much research work can be found for the analysis of FGMs plates with cutouts. Do and Lee [28] combined the isogeometric analysis method with a new quasi-3D higher-order shear deformation theory to analyze free vibration response of functionally graded material plates with complex cutouts. Bansal et al. [29] studied the porous functionally graded plate with geometric discontinuities and partial supports, the displacement field had been refined by dividing the in-plane and out of the plane displacements into bending and shear components. The geometric discontinuities had been incorporated in terms of a circular cutout of different sizes at the center of the plate. Asemi et al. [30] applied the finite element method and Rayleigh–Ritz energy formulation to analyze the static and free vibration of FGMs plate with a circular hole. Rahimabadi et al. [31] studied the free flexural vibration behavior of a centrally located circular or elliptical cutout and cracks emanating from the cutout of FGMs plates in thermal environment, the discontinuity surface was represented independent of the mesh by exploiting the partition of unity method framework. Enab et al. [32] predicted the stress concentration factors (SCFs) at the root of an elliptic hole in unidirectional functionally graded material (UDFGM) plates under uniaxial and biaxial loads by using the FEM. Janghorban et al. [33] investigated the free vibration of functionally graded non-uniform straightsided plates with circular and non-circular cutouts in a thermal environment, including the square plates, skew plates, and trapezoidal plates. Zhao et al. [34] investigated the FG plates that contain square and circular cutouts at the center, and it was found that the size of the cutout presents a considerable impact not only on the buckling loading, but also on the buckling mode shapes of the plate.

Concerning the above review of literature, it can be noticed that there are only a few papers available on the study of vibration analysis of FGMs plates with cutouts, and in the existing research, the main type of cutout is circular cutout. In addition, there is no report which investigated the vibration solution of FGMs rectangular plate with cutouts based on the three-dimensional elasticity theory. The novelty of the present paper lies in the attempt to establish a unified theoretical analysis model of the vibration characteristics of the FGMs rectangular plate with/without cutouts, and provide the three-dimensional exact solution with general boundary conditions. The material properties of FGMs plates are supposed to vary continuously along the thickness direction in power-law distributions in terms of volume fraction. The highlight of the proposed approach is that the *p*-version method and the blending function method are employed to discretize the domain and map the closed region to the computational space. All displacements of the FGMs plates are expanded in the form of standard cosine Fourier series plus auxiliary cosine series terms which can improve the convergence speed and reduce the computational complexity. The three-dimensional elasticity theory is combined with Rayleigh–Ritz method to solve the vibration problem of FGMs plate with cutouts. Numerical examples have been studied to verify the convergence, efficiency, and accuracy of the method and the predicted results have been compared with the theoretical solutions. The effects of cutout ratios, cutout positions, and cutout numbers on the natural frequencies are further explored by a parametric study in detail.

## 2. Theoretical Formulations

### 2.1. Description of the Problem

**Figure 1:**
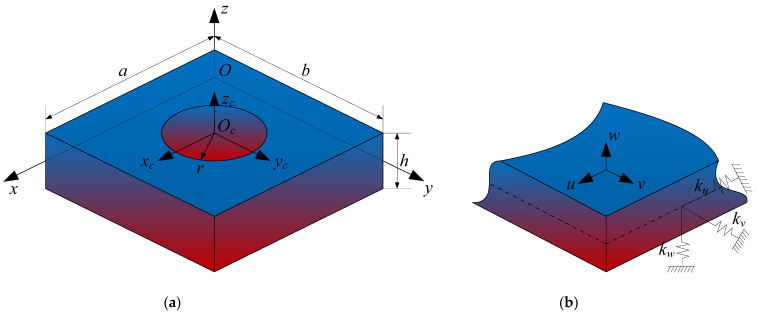
Model of a three-dimensional FGMs rectangular plate with a circular cutout: **(a)** The geometry and coordinates; **(b)** The boundary restraining springs.

In this paper, a FGMs rectangular plate with circular cutout composed of two isotropic materials is considered. Figure 1a presents the three-dimensional geometric model of the structure. In order to describe geometric model clearly, the two coordinate systems are established independently on the mid-plane of the structure, the Cartesian coordinate system (*O*-*xyz*) for the rectangular plate region which is located in the corner of the plate, and the cylindrical coordinate system (*Oc*-*x_c_y_c_z_c_*) for the cutout region which is located in the center of the circular cutout. The *x*-coordinate is taken along the length of the plate and *y*- and *z*-coordinates are taken along the width and thickness directions. The length, width, and thickness of the plate are denoted by the symbols *a*, *b*, and *h*, respectively, and the radius of the cutout is denoted by *r*. The symbols *u*, *v*, and *w* denote the displacement components in the *x*, *y*, and *z* directions, respectively.

For the FGMs composed of two types of isotropic components, such as ceramics and metals, according to the Voigt mixing rule, the equivalent materials properties can be expressed as

(1) $$E_{eff}(z)=(E_{c}-E_{m})V_{f}+E_{m}$$

(2) $$\mu_{eff}(z)=(\mu_{c}-\mu_{m})V_{f}+\mu_{m}$$

(3) $$\rho_{eff}(z)=(\rho_{c}-\rho_{m})V_{f}+\rho_{m}$$

where *E* is Young’s modulus, *μ* is Poisson’s ratio, and *ρ* is density, the subscripts *c* and *m* represents the ceramic and metal material, *V_f_* is the volume fraction.

The volume fraction, thus the variation of the material properties of each component can be obtained by assuming to be different function distributions, such as power-law functions (P-FGM), exponential functions (E-FGM), or sigmoid functions (S-FGM) [35]. In this paper, the FGMs with power-law scheme is selected as the research object.

**Figure 2:**
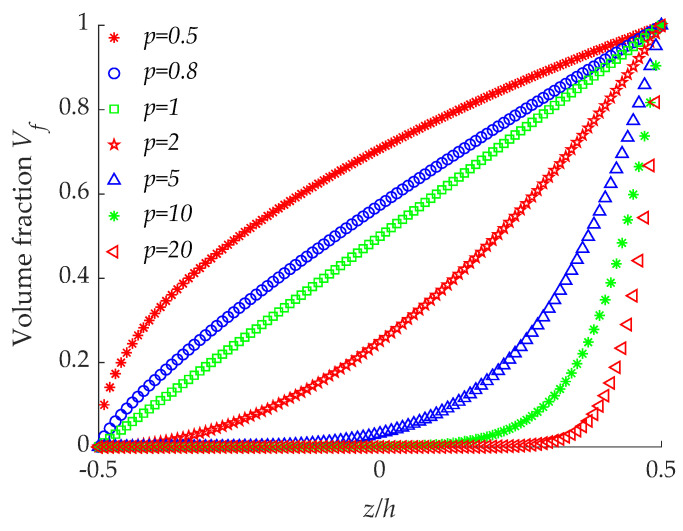
Variation of volume fraction with different gradient index.

The volume fraction *V_f_* can be defined as

(4) $$V_{f}={\left(\frac{1}{2}+\frac{z}{h}\right)}^{p},-\frac{h}{2}\leq z\leq \frac{h}{2}$$

where *z* is the thickness coordinate, and *p* is gradient index and takes only positive values. When *p* = 0, the FGMs degenerates into ceramic material and when *p* = ∞ indicates a fully metal material. Figure 2 shows the curve of volume fraction variation along the thickness direction corresponding to the different gradient index. The volume fraction can be effectively controlled by changing the value of *p*, and different kinds of FGMs can be designed by changing the above parameters according to different functional requirements.

In this paper, the FGMs plate is considered to be made of aluminum (Al) and alumina (Al_2_O_3_), the material properties for ceramic and metallic constituents of FGMs plate are listed in Table 1.

### 2.2. Kinematic Equations

The strain–displacement relations of the structure can be obtained as follows based on the linear, small-strain three-dimensional elasticity theory

(5) $$\varepsilon_{x}^{}=\frac{\partial u}{\partial x}$$

(6) $$\varepsilon_{y}^{}=\frac{\partial v^{}}{\partial y}$$

(7) $$\varepsilon_{z}^{}=\frac{\partial w^{}}{\partial z}$$

(8) $$\gamma_{yz}^{}=\frac{\partial w^{}}{\partial y}+\frac{\partial v^{}}{\partial z}$$

(9) $$\gamma_{xz}^{}=\frac{\partial u^{}}{\partial z}+\frac{\partial w^{}}{\partial x}$$

(10) $$\gamma_{xy}^{}=\frac{\partial u^{}}{\partial y}+\frac{\partial v^{}}{\partial x}$$

where *ε_x_*, *ε_y_*, *ε_z_*, *γ_yz_*, *γ_xz_*, and *γ_xy_* are the normal and shear strain.

According to the theory of three-dimensional constraint of a linear elasticity, the corresponding stress–strain relations of the three-dimensional structure are written as

(11) $$\left[\begin{array}{c} \sigma_{x}^{}\\ \sigma_{y}^{}\\ \sigma_{z}^{}\\ \tau_{yz}^{}\\ \tau_{xz}^{}\\ \tau_{xy}^{} \end{array}\right]=\left[\begin{array}{cccccc} \bar{C_{11}}(z) & \bar{C_{12}}(z) & \bar{C_{13}}(z) & 0 & 0 & \bar{C_{16}}(z)\\ \bar{C_{12}}(z) & \bar{C_{22}}(z) & \bar{C_{23}}(z) & 0 & 0 & \bar{C_{26}}(z)\\ \bar{C_{13}}(z) & \bar{C_{23}}(z) & \bar{C_{33}}(z) & 0 & 0 & \bar{C_{36}}(z)\\ 0 & 0 & 0 & \bar{C_{44}}(z) & \bar{C_{45}}(z) & 0\\ 0 & 0 & 0 & \bar{C_{45}}(z) & \bar{C_{55}}(z) & 0\\ \bar{C_{16}}(z) & \bar{C_{26}}(z) & \bar{C_{36}}(z) & 0 & 0 & \bar{C_{66}}(z) \end{array}\right]\left[\begin{array}{c} \varepsilon_{x}^{}\\ \varepsilon_{y}^{}\\ \varepsilon_{z}^{}\\ \gamma_{yz}^{}\\ \gamma_{xz}^{}\\ \gamma_{xy}^{} \end{array}\right]$$

where *C_ij_*(*z*) (*i, j* = 1, 2,…, 6) are material elastic constants, for isotropic materials, they can be defined as

(12) $$\bar{C_{11}}(z)=\bar{C_{22}}(z)=\bar{C_{33}}(z)=\frac{E_{eff}(z)(1-\mu_{eff}(z))}{\left(1+\mu_{eff}(z))(1-2\mu_{eff}(z)\right)}$$

(13) $$\bar{C_{12}}(z)=\bar{C_{13}}(z)=\bar{C_{23}}(z)=\frac{E_{eff}(z)\mu_{eff}(z)}{\left(1+\mu_{eff}(z))(1-2\mu_{eff}(z)\right)}$$

(14) $$\bar{C_{44}}(z)=\bar{C_{55}}(z)=\bar{C_{66}}(z)=\frac{E_{eff}(z)}{2(1+\mu_{eff}(z))}$$

(15) $$\bar{C_{16}}(z)=\bar{C_{26}}(z)=\bar{C_{36}}(z)=\bar{C_{45}}(z)=0$$

### 2.3. Boundary Conditions

As illustrated in Figure 1b, three groups of boundary springs are factitiously distributed along the edges to simulate different boundary conditions. The symbols *ku*, *kv*, and *kw* are used to indicate the stiffness of the springs, and through adopting appropriate values of the boundary spring stiffness, the classical boundary conditions and elastic boundary conditions can be achieved. The general boundary conditions mainly include free (F), simply supported (S), clamped (C). The expressions of the different boundary conditions along the edge *x* = 0 are given as follows.

Free boundary condition:

(16) $$\sigma_{x}=\tau_{xz}=\tau_{xy}=0$$

Simply supported boundary condition:

(17) $$\sigma_{x}=v=w=0$$

Clamped boundary condition:

(18) u=v=w=0

Elastic restraint boundary condition:

(19) u≠v≠w≠0

### 2.4. Energy Equations

In the light of Hamilton’s principle, the governing equation of the structure and the boundary conditions are derived in the following work. In this paper, the plate domain and the cutout domain are separated, and the energy functions of the FGMs plate and the cutouts are established independently. The kinetic energy function of the FGMs plate and cutouts can be expressed as

(20) $$T=\frac{1}{2}{∭}_{V}[{\left(\frac{\partial u}{\partial t}\right)}^{2}+{\left(\frac{\partial v}{\partial t}\right)}^{2}+{\left(\frac{\partial w}{\partial t}\right)}^{2}]dV$$

The total linear elastic strain energy function is depicted as

(21) $$\begin{array}{ll} U= & \frac{1}{2}{∭}_{V}\left[\sigma_{x}^{}\varepsilon_{x}^{}+\sigma_{y}^{}\varepsilon_{y}^{}+\sigma_{z}^{}\varepsilon_{z}^{}+\tau_{yz}^{}\gamma_{yz}^{}+\tau_{xz}^{}\gamma_{xz}^{}+\tau_{xy}^{}\gamma_{xy}^{}\right]dV\\ & =\frac{1}{2}{∭}_{V}\{Q_{11}(z)\left[{\left(\frac{\partial u}{\partial x}\right)}^{2}+{\left(\frac{\partial v}{\partial y}\right)}^{2}+{\left(\frac{\partial w}{\partial z}\right)}^{2}\right]+2Q_{12}(z)\left[\frac{\partial u}{\partial x}\frac{\partial v}{\partial y}+\frac{\partial u}{\partial x}\frac{\partial w}{\partial z}+\frac{\partial v}{\partial y}\frac{\partial w}{\partial z}\right]\\ & +Q_{22}(z)\left[{\left(\frac{\partial w}{\partial y}+\frac{\partial v}{\partial z}\right)}^{2}+{\left(\frac{\partial u}{\partial z}+\frac{\partial w}{\partial x}\right)}^{2}+{\left(\frac{\partial u}{\partial y}+\frac{\partial v}{\partial x}\right)}^{2}\right]\}dV \end{array}$$

The potential energy stored in the boundary springs is expressed as

(22) $$\begin{array}{ll} V= & \frac{1}{2}\iint_{yz}\left\{{\left[k_{x0}^{u}u^{2}+k_{x0}^{v}v^{2}+k_{x0}^{w}w^{2}\right]}_{x=0}+{\left[k_{xa}^{u}u^{2}+k_{xa}^{v}v^{2}+k_{xa}^{w}w^{2}\right]}_{x=a}\right\}dydz\\ & +\frac{1}{2}\iint_{xz}\left\{{\left[k_{y0}^{u}u^{2}+k_{y0}^{v}v^{2}+k_{y0}^{w}w^{2}\right]}_{y=0}+{\left[k_{yb}^{u}u^{2}+k_{yb}^{v}v^{2}+k_{yb}^{w}w^{2}\right]}_{y=b}\right\}dxdz \end{array}$$

### 2.5. Region Mapping

**Figure 3:**
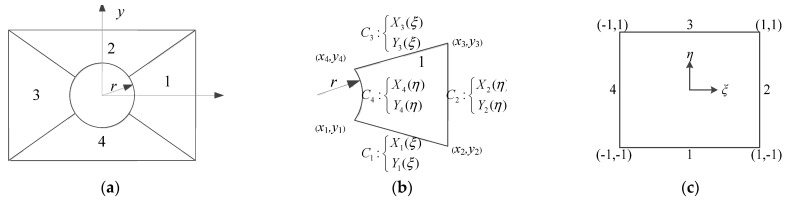
Discretization of rectangular plate with circular cutout: (**a**) geometric region; (**b**) closed quadrilateral region; (**c**) calculation region.

As described in the above model, the coordinate system of the plate is Cartesian coordinate system, and the coordinate system of the cutout is cylindrical coordinate system. The expression of the displacement components is different for the plate and cutout domain, which leads to the complexity of the energy integration. In order to simplify the solution process, a unified coordinate system is needed. The main purpose of this paper to deal with the vibration of rectangular plate with circular cutout is region mapping. The *p*-version of the finite element method is applied to discretize the plate with cutout into four curve quadrilateral sub-domains as presented in Figure 3a. The length and width of the rectangular plate are *a* and *b*, and the radius of the circular cutout is *r*, respectively. The sub-domain 1 is regarded as a closed region composed of four curves in the *x-y* coordinate system. The purpose of region mapping is to map the closed quadrilateral region into a unit square region, as described in Figure 3b,c.

The closed quadrilateral region consists of four curves, and the curve sides are in the parametric form: $C_{1}:X_{1}(\xi ),Y_{1}(\xi )$, $C_{2}:X_{2}(\eta ),Y_{2}(\eta )$, $C_{3}:X_{3}(\xi ),Y_{3}(\xi )$, $C_{4}:X_{4}(\eta ),Y_{4}(\eta )$, where −1≤ξ≤1, −1≤η≤1; the four vertex-nodes are $x_{i}$, $y_{i}$ (i=1,2,3,4), respectively. The blending function method proposed by Gordon and Hall [36] is well suited for the purpose to map the closed quadrilateral region to the computational space.

The mapping functions are given as

(23) $$\begin{array}{ll} x(\xi ,\eta ) & =(\frac{1-\eta }{2})X_{1}(\xi )+(\frac{1+\xi }{2})X_{2}(\eta )\\ & +(\frac{1+\eta }{2})X_{3}(\xi )+(\frac{1-\xi }{2})X_{4}(\eta )\\ & -[(\frac{\left(1-\xi )(1-\eta \right)}{4})x_{1}+(\frac{\left(1-\xi )(1+\eta \right)}{4})x_{2}\\ & +(\frac{\left(1+\xi )(1+\eta \right)}{4})x_{3}+(\frac{\left(1+\xi )(1-\eta \right)}{4})x_{4}] \end{array}$$

(24) $$\begin{array}{ll} y(\xi ,\eta ) & =(\frac{1-\eta }{2})Y_{1}(\xi )+(\frac{1+\xi }{2})Y_{2}(\eta )\\ & +(\frac{1+\eta }{2})Y_{3}(\xi )+(\frac{1-\xi }{2})Y_{4}(\eta )\\ & -[(\frac{\left(1-\xi )(1-\eta \right)}{4})y_{1}+(\frac{\left(1-\xi )(1+\eta \right)}{4})y_{2}\\ & +(\frac{\left(1+\xi )(1+\eta \right)}{4})y_{3}+(\frac{\left(1+\xi )(1-\eta \right)}{4})y_{4}] \end{array}$$

Substituting the circular equations and the three linear equations into Equations (23) and (24), the following results can be obtained by

(25) $$\begin{array}{ll} x(\xi ,\eta ) & =\frac{1}{4}X_{1}(\xi )+X_{2}(\eta )(\frac{\xi \eta }{4}-\frac{\eta }{4})\\ & +X_{3}(\xi )(\frac{1}{4}+\frac{\eta }{4}+\frac{\xi }{4}+\frac{\xi \eta }{4})\\ & +\frac{r\cos(\frac{\pi \eta }{4})}{2}-\frac{\xi r\cos(\frac{\pi \eta }{4})}{2} \end{array}$$

(26) $$\begin{array}{ll} y(\xi ,\eta ) & =(\frac{1-\eta }{2})Y_{1}(\xi )+(\frac{1+\xi }{2})Y_{2}(\eta )\\ & +(\frac{1+\eta }{2})Y_{3}(\xi )+(\frac{1-\xi }{2})Y_{4}(\eta )\\ & -[(\frac{\left(1-\xi )(1-\eta \right)}{4})y_{1}+(\frac{\left(1-\xi )(1+\eta \right)}{4})y_{2}\\ & +(\frac{\left(1+\xi )(1+\eta \right)}{4})y_{3}+(\frac{\left(1+\xi )(1-\eta \right)}{4})y_{4}] \end{array}$$

In the light of above region mapping and coordinate transformation relationship, the displacement components of transformation matrix from the global coordinate system to the local coordinate system are related by

(27) $$\left\{\begin{array}{c} \frac{\partial \Omega_{\left(x,y,z\right)}}{\partial x}\\ \frac{\partial \Omega_{\left(x,y,z\right)}}{\partial y}\\ \frac{\partial \Omega_{\left(x,y,z\right)}}{\partial z} \end{array}\right\}=J\left\{\begin{array}{c} \frac{\partial \Omega_{\left(\xi ,\eta ,\gamma \right)}}{\partial \xi }\\ \frac{\partial \Omega_{\left(\xi ,\eta ,\gamma \right)}}{\partial \eta }\\ \frac{\partial \Omega_{\left(\xi ,\eta ,\gamma \right)}}{\partial \gamma } \end{array}\right\}$$

where *J* is the Jacobian transformation matrix, and the mathematical expression of Jacobian matrix is

(28) $$J=\left[\begin{array}{ccc} J_{1,1} & J_{1,2} & J_{1,3}\\ J_{2,1} & J_{2,2} & J_{2,3}\\ J_{3,1} & J_{3,2} & J_{3,3} \end{array}\right]=\left[\begin{array}{ccc} \frac{\partial x_{\left(\xi ,\eta ,\gamma \right)}}{\partial \xi } & \frac{\partial y_{\left(\xi ,\eta ,\gamma \right)}}{\partial \xi } & \frac{\partial z_{\left(\xi ,\eta ,\gamma \right)}}{\partial \xi }\\ \frac{\partial x_{\left(\xi ,\eta ,\gamma \right)}}{\partial \eta } & \frac{\partial y_{\left(\xi ,\eta ,\gamma \right)}}{\partial \eta } & \frac{\partial z_{\left(\xi ,\eta ,\gamma \right)}}{\partial \eta }\\ \frac{\partial x_{\left(\xi ,\eta ,\gamma \right)}}{\partial \gamma } & \frac{\partial y_{\left(\xi ,\eta ,\gamma \right)}}{\partial \gamma } & \frac{\partial z_{\left(\xi ,\eta ,\gamma \right)}}{\partial \gamma } \end{array}\right]$$

The inverse of the Jacobian transformation matrix is

(29) $$J^{-1}=\frac{1}{\left|J\right|}J^{*}$$

(30) $$\begin{array}{l} \left|J\right|=J_{1,1}J_{2,2}J_{3,3}+J_{1,2}J_{2,3}J_{3,1}+J_{1,3}J_{2,1}J_{3,2}\\ -J_{1,1}J_{2,3}J_{3,2}-J_{1,2}J_{2,1}J_{3,3}-J_{1,3}J_{2,2}J_{3,1} \end{array}$$

(31) $$J^{*}=\left[\begin{array}{ccc} J_{2,2}J_{3,3}-J_{2,3}J_{3,2} & J_{2,3}J_{3,1}-J_{2,1}J_{3,3} & J_{2,1}J_{3,2}-J_{2,2}J_{3,1}\\ J_{1,3}J_{3,2}-J_{1,2}J_{3,3} & J_{1,1}J_{3,3}-J_{1,3}J_{3,1} & J_{1,2}J_{3,1}-J_{1,1}J_{3,2}\\ J_{1,2}J_{2,3}-J_{1,3}J_{2,2} & J_{1,3}J_{2,1}-J_{1,1}J_{2,3} & J_{1,1}J_{2,2}-J_{1,2}J_{2,1} \end{array}\right]$$

### 2.6. Solution Procedure

In this paper, the Rayleigh–Ritz method is used due to it is applicable to arbitrary boundary conditions without requiring any special procedures. Thus, it is very important to construct an admissible displacement function field because the accuracy and the convergence of the solution depend on the accuracy of the expression of the admissible displacement function. In this paper, the improved Fourier series method is further extended to the three-dimensional vibration analysis of FGMs rectangular plate with cutouts. According to the author’s previous research, the admissible displacement functions are consistent with [37], and expressed in the form of complete trigonometric Fourier series, thus the auxiliary terms are also in the form of trigonometric Fourier series. The three-dimensional admissible displacement functions of the FGMs plate are expressed as three variables separated along the *x*, *y*, and *z* directions as

(32) $$\begin{array}{ll} U(x,y,z)= & \sum_{m=0}^{M}\sum_{n=0}^{N}\sum_{q=0}^{Q}A_{mnq}\cos\lambda_{m}x\cos\lambda_{n}y\cos\lambda_{q}z\\ & +\sum_{m=0}^{M}\sum_{n=0}^{N}\left[a_{u\_z}\xi_{1c}(z)+b_{u\_z}\xi_{2c}(z)\right]\cos\lambda_{m}x\cos\lambda_{n}y\;\\ & +\sum_{m=0}^{M}\sum_{q=0}^{Q}\left[a_{u\_y}\xi_{1b}(y)+b_{u\_y}\xi_{2b}(y)\right]\cos\lambda_{m}x\cos\lambda_{q}z\\ & +\sum_{n=0}^{N}\sum_{q=0}^{Q}\left[a_{u\_x}\xi_{1a}(x)+b_{u\_x}\xi_{2a}(x)\right]\cos\lambda_{n}y\cos\lambda_{q}z \end{array}$$

(33) $$\begin{array}{ll} V(x,y,z)= & \sum_{m=0}^{M}\sum_{n=0}^{N}\sum_{q=0}^{Q}B_{mnq}\cos\lambda_{m}x\cos\lambda_{n}y\cos\lambda_{q}z\\ & +\sum_{m=0}^{M}\sum_{n=0}^{N}\left[a_{v\_z}\xi_{1c}(z)+b_{v\_z}\xi_{2c}(z)\right]\cos\lambda_{m}x\cos\lambda_{n}y\;\\ & +\sum_{m=0}^{M}\sum_{q=0}^{Q}\left[a_{v\_y}\xi_{1b}(y)+b_{v\_y}\xi_{2b}(y)\right]\cos\lambda_{m}x\cos\lambda_{q}z\\ & +\sum_{n=0}^{N}\sum_{q=0}^{Q}\left[a_{v\_x}\xi_{1a}(x)+b_{v\_x}\xi_{2a}(x)\right]\cos\lambda_{n}y\cos\lambda_{q}z \end{array}$$

(34) $$\begin{array}{ll} W(x,y,z)= & \sum_{m=0}^{M}\sum_{n=0}^{N}\sum_{q=0}^{Q}C_{mnq}\cos\lambda_{m}x\cos\lambda_{n}y\cos\lambda_{q}z\\ & +\sum_{m=0}^{M}\sum_{n=0}^{N}\left[a_{w\_z}\xi_{1c}(z)+b_{w\_z}\xi_{2c}(z)\right]\cos\lambda_{m}x\cos\lambda_{n}y\;\\ & +\sum_{m=0}^{M}\sum_{q=0}^{Q}\left[a_{w\_y}\xi_{1b}(y)+b_{w\_y}\xi_{2b}(y)\right]\cos\lambda_{m}x\cos\lambda_{q}z\\ & +\sum_{n=0}^{N}\sum_{q=0}^{Q}\left[a_{w\_x}\xi_{1a}(x)+b_{w\_x}\xi_{2a}(x)\right]\cos\lambda_{n}y\cos\lambda_{q}z \end{array}$$

where $\lambda_{m}=m\pi /a$, $\lambda_{n}=n\pi /b$, $\lambda_{q}=q\pi /h$, and $A_{mnq}$, $B_{mnq}$, $C_{mnq}$, $a_{u}$, $a_{v}$, $a_{w}$, $b_{u}$, $b_{v}$, $b_{w}$ are the unknown Fourier coefficients, *M*, *N*, and *Q* are the truncation numbers with respect to variables *x*, *y*, and *z* directions, respectively. In order to unify the form of the admissible displacement functions and simplify the mathematical processing, the supplementary functions are defined as

(35) $$\xi_{1a}(x)=\frac{1}{2}\left[\sin(2\pi x/a)+\sin(\pi x/a)\right]$$

(36) $$\xi_{2a}(x)=\frac{1}{2}\left[\cos(3\pi x/2a)-\cos(\pi x/2a)\right]$$

(37) $$\xi_{1b}(y)=\frac{1}{2}\left[\sin(2\pi y/b)+\sin(\pi y/b)\right]$$

(38) $$\xi_{2b}(y)=\frac{1}{2}\left[\cos(3\pi y/2b)-\cos(\pi y/2b)\right]$$

(39) $$\xi_{1c}(z)=\frac{1}{2}\left[\sin(2\pi z/h)+\sin(\pi z/h)\right]$$

(40) $$\xi_{2c}(z)=\frac{1}{2}\left[\cos(3\pi z/2h)-\cos(\pi z/2h)\right]$$

The total energy of the FGMs rectangular plate with circular cutout is defined by subtracting the energy of cutout domain part from the entire plate domain. Thus, the Lagrangian energy function of the structure can be expressed as

(41) $$\prod =\prod_{p}-\sum_{i=1}^{n}\prod_{i}$$

The subscripts “*p*” and “*i*” denote the energy of the plate and cutout domains, respectively.

For the plate without cutout, the energy equation is

(42) $$\prod =T_{p}-U_{p}-V_{p}$$

Substituting Equations (20)–(22), (27), and (32)–(34) and into Equation (41), and by minimizing the Lagrangian energy functional ∏ with respect to each unknown coefficients to be zero, we can get the equation

(43) $$\frac{\partial \prod }{\partial X}=0(X=A_{mnq},B_{mnq},C_{mnq},a_{u},a_{v},a_{w},b_{u},b_{v},b_{w})$$

The standard eigenvalue equation of motion for rectangular plate with circular cutout can be expressed in the form of matrix

(44) $$[(K_{p}-\sum_{i=1}^{n}K_{i})-\omega^{2}(M_{p}-\sum_{i=1}^{n}M_{i})]X=(K-\omega^{2}M)X=0$$

where K, M, and X are the stiffness matrices, mass matrices and the unknown Fourier coefficients matrices, respectively. All of the natural frequencies and mode shapes of the three-dimensional FGMs rectangular plates with circular cutouts can be obtained by solving Equation (44).

## 3. Results and Discussion

In this section, according to the unified theoretical analysis model established above, several examples for the three-dimensional vibration analysis of FGMs plate with/without cutouts are presented to illustrate the accuracy and reliability of the proposed method. Firstly, a suitable spring stiffness value is investigated, and then the convergence, efficiency and validation are checked. Secondly, the vibration modal experiment of an aluminum square plate with a center circular cutout is conducted to verify the correctness of the proposed method. Finally, a parametric study of the FGMs plate with cutouts is carried out from free vibration characteristics and harmonic response analysis, including the cutout sizes, cutout positions, and cutout numbers.

For simplicity, the boundary conditions of the structure are described in the form of character combination, unless other stated, the non-dimensional frequency parameter is expressed as: $\Omega =\omega a^{2}\sqrt{\rho_{c}h/D_{c}}$, where $D_{c}$ is the flexural stiffness of Alumina, $D_{c}=E_{c}h^{3}/12(1-\mu^{2})$.

### 3.1. Determination of the Spring Stiffness

**Figure 4:**
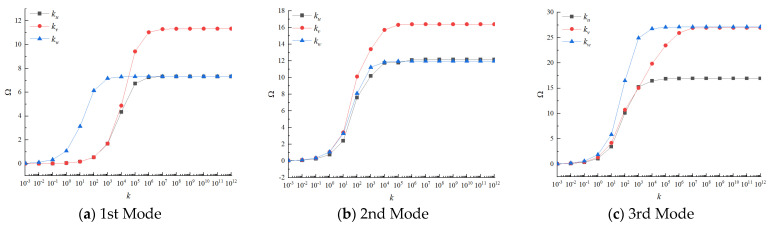
Variation of non-dimensional frequency parameters versus different spring stiffness for FGMs rectangular plate.

In this paper, three groups of linear springs are introduced to simulate different kinds of boundary conditions by changing the values of spring stiffness. The accuracy of the solutions is strongly affected by the selection of appropriate spring stiffness values. Therefore, in this section, the FGMs square plate without cutout is taken as an example to study the determination of the spring stiffness. The variations of the first three non-dimensional frequency parameters of FGMs square plate versus different spring stiffness are given in Figure 4. The geometric dimensions and the material parameters are as follows: a=b=1 m, h=0.05 m, and p=1. The boundary conditions of the plate are defined as: the edge *y* = 0 and *y* = b are completely free and the *x* = 0 and *x* = a are elastic supported by one group of spring constrain varying from 10^−3^*D_c_* to 10^12^*D_c_*. From Figure 4, it can be seen that the non-dimension frequency parameters are unchanged and approaches 0 when the spring stiffness is smaller than 10^−1^*D_c_*, and when the spring stiffness is varied from 10^−1^*D_c_* to 10^7^*D_c_*, the frequency parameters increase rapidly. Finally, when the spring stiffness exceeds 10^7^*D_c_*, the frequency parameters will approach their utmost and tend to be stable. Therefore, we can arrive at the conclusion that the free boundary conditions and clamped boundary conditions can be simulated by assigning the spring stiffness value to be 0 or 10^7^*D_c._*

### 3.2. Convergence of the Method

Convergence property for the free vibration analysis of FGMs plate with circular cutout is examined in terms of the limited number of terms in the displacement expressions in actual calculation to verify the accuracy and efficiency of the proposed method. Table 2 shows the convergence studies of the first six non-dimensional frequency parameters of a FGMs square plate with a central circular cutout with different truncated numbers, and the data of [38] are also given out in Table 2. The truncated number of admissible displacement function components in Fourier series expansion is expressed as *M* × *N* × *Q*, and the truncated number of this paper is from 3 to 10. It can be observed that the maximum error with [38] is 1.7898%, and the main reason of the error is that the first order shear theory is adopted by [38]. It can be concluded that the proposed method has fast convergence and good stability, and the truncation numbers will be set as *M* = *N* = *Q =* 10 in the following studies.

### 3.3. Validity of the Method

#### 3.3.1. Numerical Examples Study

In this section, in order to verify the proposed method is also suitable for solving the vibration characteristics of FGMs plate without cutouts, the comparison study of a FGMs square plate under SSSS boundary condition will be carried out by the present method and other method presented in [25]. Table 3 shows the first eight non-dimensional frequency parameters of FGMs square plate which was studied by Huang et al. The values of the gradient indexes are taken to be 0, 1, 2, 5, 10. The symbol ‘-’ indicates that the frequencies were not considered in the reference work. In the table, the thickness–length ratio is taken as 0.1 and 0.2, it is moderate–thick plate structure. From the comparison, we can see a consistent agreement of the results taken from the current method and the referential data. From the tables, it is obvious that these data show a similar behavior, that is the frequency parameters decrease with the increase of gradient index. When the thickness–length ratio is 0.1, the gradient index increases from 0 to 10, the first-order non-dimensional frequency parameter decreases by 36.95%, and when the thickness–length ratio is 0.1, the first-order non-dimensional frequency parameter decline rate is 38.44%. The main reason is that the increase of gradient index leads to the decrease of the volume fraction of the corresponding ceramic components, which reduces the stiffness of the structure, and finally leads to the decrease of frequency. In addition, the increase of thickness–length ratios leads to decrease of the frequency parameters for all of the cases considered. By way of illustration, as the thickness–length ratio increases, the first-order non-dimensional frequency parameter drops by 8.19% when the gradient index is 0. According to the relationship between the non-dimensional frequency parameters calculation formula and thickness, it can be known that the natural frequencies increase with the increase of thickness. The increase of thickness will increase the stiffness and mass of rectangular plate, but the increase of stiffness plays a decisive role in the influence of natural frequency.

For the next comparison study, a FGMs rectangular plate with a central circular cutout under CCCC-F boundary condition is examined. In Table 4, the first fix non-dimensional frequency parameters are obtained. It is observed that the frequencies are in excellent agreement with those given in [38], which verifies the accuracy and efficiency of the proposed model. The effect of gradient index on the frequency parameters of structure with cutout is consistent with that structure without cutout also can be concluded from the table below. Therefore, the effect of material parameters on structure with cutout is independent of the geometric size of the structure. In addition, it can be seen that the frequency parameters show an increasing trend with the increase of the structural aspect ratio, but it is not a linear trend. Take the gradient index is 0 as an example, when the aspect ratio is 1.5, the first-order non-dimensional frequency parameter is 1.67 times that when the aspect ratio is 1, while it is 2.7 times when the aspect ratio is 2. This is mainly because when the aspect ratio increases, the overall mass and stiffness of the structure will also increase, and the stiffness is the main factor affecting frequency parameters.

Numerous results of the first six non-dimensional frequency parameters are demonstrated in Table 5 for the FGMs square plate with central circular cutout under different boundary conditions. The square plate with boundary restrains, including SSSS-F, SCSC-F, SFSF-F, FCFC-F, FFCF-F, FCCC-F, FSCS-F, and FCCF-F are considered. From the table below, the influence of boundary conditions on the frequency parameters is obvious, the stronger the boundary restrains, the higher the corresponding frequency parameters, this can be clearly confirmed from SFSF-F, SSSS-F, and SCSC-F boundary conditions. Comparing three groups of boundary conditions—FCFC-F, FCCF-F, and FCCC-F—it can be concluded that the clamped boundary constrain plays an important role in the frequency parameters, while frequency parameter of the opposite side constraint is larger than that of the adjacent side constraint.

**Figure 5:**
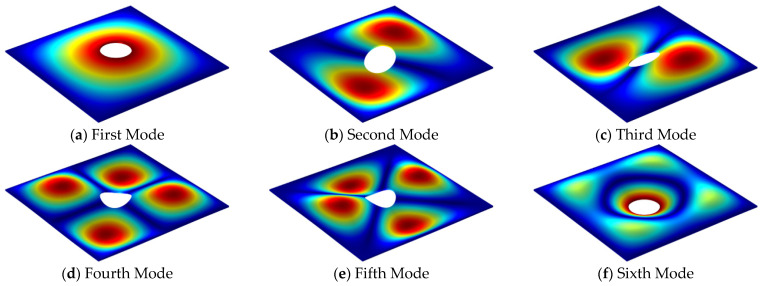
The first six mode shapes for SSSS-F FGMs square plate with central circular cutout (*h/a* = 0.01, *p* = 1).

**Figure 6:**
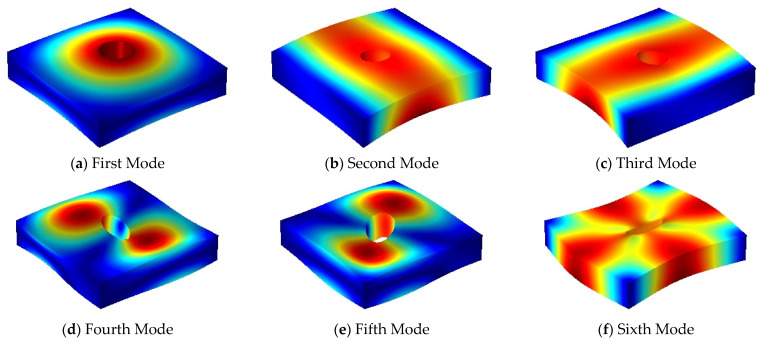
The first six mode shapes for SSSS-F FGMs square plate with central circular cutout (*h/a* = 0.2, *p* = 1).

The first six mode shapes of the FGMs square plate with central circular cutout under SSSS-F boundary condition when the thickness–length ratio is 0.01 and 0.2 are presented in Figure 5 and Figure 6, respectively. From the graphs below, for thin plate structure, the lower order mode shapes are mainly transverse vibration, while for moderate thick plate structure, the shear deformation along the thickness direction gradually appears. The three dimensions of the elastic plate structure have complex deformation forms for different modes, therefore the analysis based on the three-dimensional elastic theory can fully consider the influence of the shear deformation in the thickness direction on the vibration characteristics of the structure.

#### 3.3.2. Experimental Study

**Figure 7:**
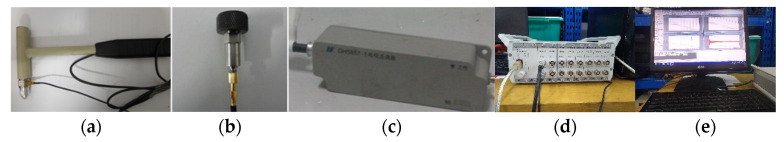
The experimental setup: (**a**) hammer; (**b**) accelerometer sensor; (**c**) charge adapter; (**d**) dynamic data acquisition instrument; (**e**) computer.

**Figure 8:**
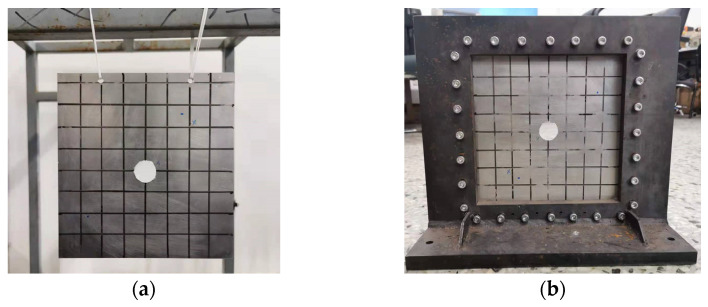
The FGMs plate with central circular cutout: (**a**) FFFF-F boundary condition; (**b**) CCCC-F boundary condition.

In this part, an experimental study of plate with central cutout is conducted to further verify the validity of the proposed method. The experimental setup—including the hammer, accelerometer sensor, charge adapter, dynamic data acquisition instrument, and computer—is shown in Figure 7. In view of the available situation, the gradient index is considered to be infinity, thus the functionally graded material degenerates to be completely aluminum. A square plate with central circular cutout under FFFF-F and CCCC-F boundary condition are examined. It is impossible to realize the complete free boundary condition in the actual experimental environment, two small holes are opened on the edge of the structure, and elastic rubber ropes are used to hang the structure on the frame, as shown in Figure 8a. For the CCCC-F boundary condition, the experimental model adopts two thicker L-shaped plates and arranges bolts uniformly around the rectangular plate structure to simulate the fixed boundary condition, as shown in Figure 8b. The parameters of dimension and the material of the structure are given in Table 6. Table 7 shows the first six natural frequencies of the structure obtained by the present method and the experiment. Through the comparative analysis of experiments and the calculation of the proposed method, the difference is 4.754% for the worst case, which is acceptable. The main reason for the error lies in two aspects. Firstly, the difference of boundary restrains between the experimental simulation and the theoretical calculation will cause a certain error. Secondly, when knocking with a hammer, it requires that the knocking direction is completely perpendicular to the panel surface, the knocking force should be constant, and the hammer shall be evacuated quickly when the knocking is finished to avoid secondary knocking, which is difficult to ensure in the process of experiment. The experimental frequency values are obtained from the vibration analysis software by searching the peak within a certain range of the frequency, and three are some interference items near desired the frequency value, the peak value is automatically identified and selected by the computer, this is also the reason for the error. The experimental values are smaller than the theoretical value, the main reason is due to the additional mass of the accelerometer which is attached to the panel.

### 3.4. Parametric Study

In this section, the parametric study of three-dimensional vibration characteristics of the FGMs plate with cutouts is carried out. Based on the existing literature, the structural vibration characteristics of different functionally graded material parameters are different, and the predecessors have done a lot of research on this. This section emphasizes the study of the influence of the parameters of the cutout on the free vibration characteristics and harmonic response analysis of the structure, including the cutout sizes, cutout positions, and number of the cutout. The FGMs square plate with circular cutouts under CCCC-F boundary conditions is taken as the analysis object, and the geometric parameters and materials parameters are set as follows: a=b=1 m, h=0.1 m, p=1.

First, the variation of the non-dimensional frequency parameters with respect to diverse cutout sizes is investigated. Table 8 presents the first six frequency parameters of the FGMs square plate with a central circular cutout, and the cutout size ratios (*r/a*) vary from 0 to 0.25. For the small values of cutout size ratio, the frequency parameters of the structure with and without cutout are almost the same. It is found that the change trend of the low-order modal frequency parameters of the structure is relatively simple, a minimum value for the first five modes exists and the frequency parameters first decrease and then increase when the cutout ratio rise, while the change of the high-order frequency parameters is more complicated. The reason may be due to the weight of mass loss and stiffness loss on the frequency parameters is different with the increase of cutout size ratio.

**Figure 9:**
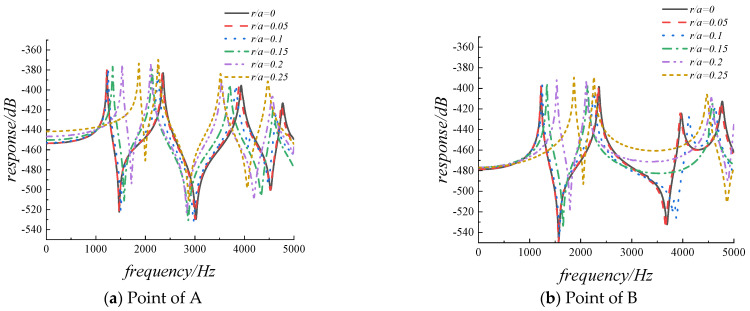
The displacement response for FGMs square plate with diverse cutout sizes.

Then, the harmonic analysis is used to analyze the steady-state response of the FGMs plate with cutouts under simple harmonic excitation. In order to overcome the problem of numerical instability caused by structural resonance at the modal frequency of the external excitation, the damping factor will be introduced in the form of complex Young’s modulus, thus $\bar{E}=E(1+j\eta )$, η=0.01. Assuming that a simple harmonic force is applied to point A along the z-axis, and the magnitude of the force is 1 N. The coordinates of the excitation force application point A and the selected response observation point B are (0.5 m, 0.8 m, 0.05 m) and (0.8 m, 0.8 m, 0.05 m), respectively. Figure 9a,b provide the results obtained from the preliminary analysis of the displacement response curve of the excitation force application point and the response observation point with frequency in the range of 0–5000 Hz. The range of the cutout size ratio is from 0 to 0.25 with a step of 0.025, and the displacement response is H=20*log(w). From the graph below we can see that there has been a slight rise in the displacement response with the gradual increase of the cutout size rate. This is mainly because the stiffness of the excitation force application point and the observation point is weakened by the introduction of the cutout. The resonance peak of the displacement response will shift left and right with the increase of cutout size ratio. To further explain, in Figure 9a, the first-order resonance peak frequency is 1225 Hz when the structure is without cutout, the first-order resonance peak frequency is 1221 Hz when the cutout ratio is 0.05, and the first-order resonance peak frequency is 1246 Hz when the cutout ratio is 0.1, in the case of a larger cutout ratio, it can be clearly seen that the first-order resonance peak frequency is increasing, which is consistent with the change trend of the data in Table 8.

The following part of the study is concerned with the position of the cutout. The radius of the cutout is 0.1 m, and the position of cutout varies along the *x*-axis. The table below illustrates the first six non-dimensional frequency parameters of the FGMs square plate with different cutout positions. In Table 9, when the cutout position $x_{c}=0.5$, it means that the cutout is located in the center of rectangular plate. The table reveals that as the cutout position gradually approaches the edge of the structure, the fundamental frequency parameter of the structure gradually declines, the second order frequency parameters of the structure gradually increases, while the higher order frequency parameters changes are more complicated.

**Figure 10:**
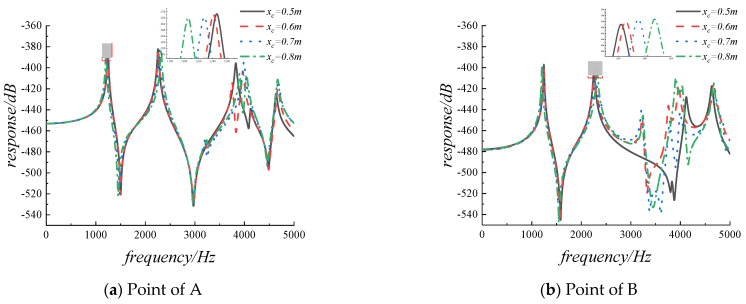
The displacement response for FGMs square plate with diverse cutout positions.

The results of the correlational analysis of displacement response for FGMs square plate with different cutout positions are shown in Figure 10. From the graph below we can see that in the frequency range of 0–3000 Hz, the vibration displacement response at the resonance peak changes slightly, for the excitation force application point, the amplitudes of the first-order resonance peaks are −378.658 dB, −378.957 dB, −379.589 dB, and −379.962 dB, respectively. While the excitation frequency is greater than 3000 Hz, the vibration displacement response at the resonance peak changes obviously.

**Figure 11:**
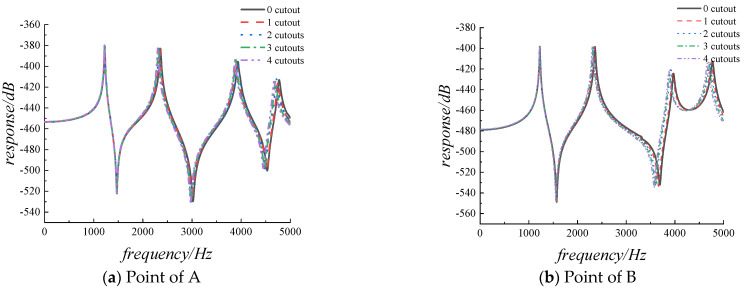
The displacement response for FGMs square plate with different cutout numbers.

In actual engineering structures, it is often necessary to evenly arrange multiple cutouts on the structure, so in the final part of the study, the influence of the number of cutout on the vibration characteristics of the structure is investigated. Table 9 provides the first six non-dimensional frequency parameters of the FGMs square plate with different cutout numbers, the cutouts are evenly distributed along the *x*-axis direction. The radius of the cutouts is 0.05 m, and the other parameters remain the same with the previous data. What can be clearly seen in Table 10 is the decrease with the increasing in the number of cutouts for the frequency parameters of all orders. The results of the harmonic response correlational analysis of are presented in Figure 11. The graph shows that there has been a small change for the amplitude of the displacement response with the increasing of the cutout numbers, while all resonance peaks gradually shift to the left.

## 4. Conclusions

The aim of the present research is to establish a unified three-dimensional solution to deal with the vibration characteristics of FGMs plate with/without circular cutouts. The material properties vary continuously along the thickness direction according to the power-law distribution. The artificial spring technology is used to simulate the general boundary conditions by setting three groups of linear springs and assigning them with appropriate spring stiffness values. Due to relatively complicated governing differential equations and domain of the problem, the *p*-version of the finite element method is applied to discretize the plate with cutout into four curve quadrilateral sub-domains, and then map the closed quadrilateral region to the computational space by the blending function method. The independent coordinate coupling relationship is used to derive the Jacobian relationship matrix of the rectangular plate domain and the circular cutout domain, finally the Lagrangian energy equation is used to solve the differential equation. In the analysis of numerical examples, it is found that the calculation results of this method are in good agreement with other results through comparison with the existing literature and finite element simulation analysis results, which verifies that the method proposed in this paper is reliable. Then the effects of cutout sizes, cutout positions, and cutout numbers on the frequency parameters of FGMs plate with cutout are studied and discussed, and all of these factors will have an impact on the frequency parameters. The proposed method can be applicable to solve the vibration of complex shape plate with cutouts, and the numerical results can be useful for future research.

## Figures and Tables

**Table 1:** Material properties of the FGMs plate.

Properties	Aluminum (Al)	Alumina (Al2O3)	Unit
E	70	380	GPa
ρ	2700	3800	Kg/m^3^
μ	0.3	0.3	

**Table 2:** Non-dimensional frequency parameters of FGMs square plate with a central circular cutout under FFFF-F boundary condition ($\Omega =\omega a^{2}\sqrt{\rho_{c}h/E_{c}}$, a=b=1 m, r=0.1 m, h=0.01 m, p=1).

*M* × *N* × *Q*	Modes					
	1	2	3	4	5	6
3 × 3 × 3	10.1684	14.8912	18.6946	27.6210	27.7461	49.5969
4 × 4 × 4	10.0023	14.4524	17.7520	26.5533	26.5588	46.3665
5 × 5 × 5	9.9825	14.4184	17.6390	26.4671	26.4685	46.0454
6 × 6 × 6	9.9729	14.4053	17.5977	26.4291	26.4292	45.9154
7 × 7 × 7	9.9640	14.3993	17.5772	26.4032	26.4036	45.8507
8 × 8 × 8	9.9589	14.3969	17.5719	26.3910	26.3912	45.8319
9 × 9 × 9	9.9597	14.3961	17.5695	26.3918	26.3923	45.8232
10 × 10 × 10	9.9564	14.3944	17.5670	26.3846	26.3849	45.8131
Ref. [38]	9.9070	14.4660	17.8200	26.4110	26.4120	46.6480
Error(%)	0.4984	0.4947	1.4196	0.1001	0.1028	1.7898

**Table 3:** The first eight non-dimensional frequency parameters of FGMs square plate without cutout under SSSS boundary condition ($\Omega =\omega a^{2}/h\sqrt{\rho_{c}/E_{c}}$, a=b=1 m).

*h/a*	*p*	Methods	Modes							
			1	2	3	4	5	6	7	8
0.1	0	Present	5.7771	13.8062	13.8062	19.4833	19.4833	21.2174	25.8742	25.8742
		Ref. [25]	5.7770	13.8100	13.8100	19.4800	19.4800	-	-	-
	1	Present	4.4267	10.6291	10.6291	16.2033	16.2033	16.4006	20.0477	20.0478
		Ref. [25]	4.4260	10.6300	10.6300	16.2000	16.2000	-	-	-
	5	Present	3.7728	8.9308	8.9308	12.6410	12.6410	13.6246	16.5482	16.5482
		Ref. [25]	3.7720	8.9270	8.9270	12.6400	12.6400	-	-	-
	10	Present	3.6424	8.5886	8.5886	11.5282	11.5282	13.0602	15.8326	15.8326
		Ref. [25]	3.6410	8.5870	8.5870	11.5200	11.5200	-	-	-
0.2	0	Present	5.3037	9.7417	9.7417	11.6456	11.6456	13.7768	16.8826	19.4833
		Ref. [25]	5.3040	9.7420	9.7420	11.6500	11.6500	-	-	-
	1	Present	4.0996	8.0899	8.0899	9.1088	9.1088	11.4184	13.3121	15.8149
		Ref. [25]	4.0990	8.0890	8.0890	9.1070	9.1070	-	-	-
	5	Present	3.4057	6.2979	6.2979	7.3454	7.3454	8.8643	10.5497	12.4190
		Ref. [25]	3.4050	6.2960	6.2960	7.3430	7.3430	-	-	-
	10	Present	3.2647	5.7508	5.7508	6.9751	6.9751	8.1082	9.9525	11.3994
		Ref. [25]	3.2640	5.7490	5.7490	6.9750	6.9750	-	-	-

**Table 4:** The first six non-dimensional frequency parameters of FGMs rectangular plate with central circular cutout under CCCC-F boundary condition (b=1 m, r=0.1 m, h=0.01 m).

*a/b*	*p*	Methods	Modes					
			1	2	3	4	5	6
1	0	Present	36.5358	71.4352	71.4386	105.4876	127.5262	138.6374
		Ref. [38]	36.4200	71.1900	71.1900	105.1500	127.0900	138.1200
	0.5	Present	31.1424	60.8977	60.9032	89.9195	108.7118	118.1773
		Ref. [38]	31.2000	61.0800	61.0800	87.5700	106.3200	116.2600
	1	Present	27.8990	54.5626	54.5691	80.5628	97.4015	105.8843
		Ref. [38]	28.1200	55.0500	55.0500	78.9700	95.8300	104.7800
	2	Present	25.3673	49.6041	49.6103	73.2430	88.5495	96.2584
		Ref. [38]	25.5700	50.0500	50.0500	71.7600	87.1200	95.2600
	10	Present	22.6006	44.1827	44.1910	65.2574	78.8860	85.7766
		Ref. [38]	23.4400	45.8800	45.8900	65.7700	79.8800	87.3300
1.5	0	Present	61.1085	92.9936	146.0148	149.5540	177.0082	221.1563
		Ref. [38]	60.9700	92.7900	145.6600	149.2100	176.5700	220.6000
	0.5	Present	52.0759	79.2583	124.4491	127.4684	150.8639	188.5330
		Ref. [38]	52.1800	78.3200	123.7200	126.1200	147.0100	187.3000
	1	Present	46.6604	71.0142	111.5217	114.2040	135.1729	168.9314
		Ref. [38]	47.0300	70.5900	111.5100	113.6600	132.5000	168.8900
	2	Present	42.4268	64.5684	101.3901	103.8366	122.8967	153.5787
		Ref. [38]	42.7500	64.1800	101.3800	103.3300	120.4500	153.5300
	10	Present	38.2502	58.1810	91.3882	93.5478	110.7397	138.2982
		Ref. [38]	39.2200	58.8500	92.9500	94.8000	110.4300	140.6000
2	0	Present	98.7524	126.9054	178.8877	250.1018	252.4322	282.2987
		Ref. [38]	98.5100	126.6400	178.5500	249.6700	251.7700	281.5500
	0.5	Present	84.1596	108.1565	152.4540	213.1582	215.1543	240.6124
		Ref. [38]	83.9400	106.4700	151.3800	210.3000	212.7000	234.3100
	1	Present	75.4040	96.9035	136.5945	190.9989	192.7910	215.5763
		Ref. [38]	75.6500	95.9500	136.4300	189.5400	191.7000	211.1900
	2	Present	68.5630	88.1106	124.1989	173.6566	175.2810	196.0024
		Ref. [38]	68.7700	87.2400	124.0300	172.3300	174.2800	191.9900
	10	Present	61.7974	79.4025	111.9418	156.5118	157.9518	176.5748
		Ref. [38]	63.1300	80.0100	113.8000	157.9200	159.8000	176.0800

**Table 5:** The first six non-dimensional frequency parameters of the FGMs square plate with central circular cutout under different boundary conditions (a=b=1 m, r=0.1 m, h=0.2 m).

*p*	Modes	BC							
		SSSS-F	SCSC-F	SFSF-F	FCFC-F	FFCF-F	FCCC-F	FSCS-F	FCCF-F
1	1	13.3179	17.9178	6.7214	14.0532	2.5402	14.8664	8.7123	4.7443
	2	27.4857	27.5032	10.8235	15.7097	5.5009	22.8422	12.9261	14.5219
	3	27.4857	30.1875	20.8144	24.0629	8.7486	31.9077	19.9885	16.5373
	4	28.7276	34.5120	21.9500	25.0945	13.3916	32.4554	25.2352	20.8138
	5	28.7276	44.1081	23.9487	31.6279	16.8760	37.6186	26.8349	26.2324
	6	31.7165	46.3976	24.1150	34.1495	18.5060	38.5433	34.1060	26.5989
2	1	12.0147	16.0673	6.0818	12.6100	2.3036	13.3293	7.8648	4.2878
	2	24.7467	24.7801	9.7578	14.0563	4.9551	20.3974	11.6737	12.9999
	3	24.7467	27.0203	18.7829	21.4707	7.9140	28.7278	17.9049	14.8286
	4	25.7677	30.7681	19.6557	22.6303	12.0132	28.9185	22.6299	18.8357
	5	25.7677	39.6501	21.4900	28.1874	15.1534	33.6924	24.2132	23.4904
	6	28.5742	41.3629	21.7518	30.3788	16.5759	34.2556	30.4527	23.9566
5	1	11.1106	14.4756	5.6752	11.3425	2.1641	11.9720	7.2773	3.9791
	2	21.3820	21.3939	9.0126	12.5809	4.5625	18.2732	10.0846	11.7849
	3	21.3820	24.0287	16.2282	19.3610	6.8440	24.8314	16.2413	13.4594
	4	23.0528	26.9232	17.9878	19.5523	10.9596	25.3154	20.4136	16.2938
	5	23.0529	34.1756	18.7834	24.6607	13.9533	29.7157	20.9315	20.5253
	6	24.6644	36.2624	19.4196	26.6505	14.9960	29.9962	27.1231	21.2598
10	1	10.6718	13.7473	5.4669	10.7539	2.0888	11.3491	6.9882	3.8236
	2	19.5447	19.5491	8.6564	11.9249	4.3773	17.3586	9.1971	11.2591
	3	19.5447	22.6779	14.8131	17.8576	6.2340	22.7150	15.5103	12.8460
	4	21.8196	25.2270	17.1491	18.4902	10.4910	23.7362	19.1146	14.8380
	5	21.8197	31.2776	17.2891	23.1096	13.4264	27.8644	19.4074	18.7537
	6	22.5428	33.9918	18.4730	25.0619	14.2976	28.1971	25.6807	20.1745

**Table 6:** Dimensions and material parameters of the structure.

Parameter	Value	Unit	Parameter	Value	Unit
length	245	mm	E	70	Gpa
width	245	mm	ρ	2700	Kg/m^3^
thickness	5	mm	μ	0.3	
radius	15	mm			

**Table 7:** The first six natural frequencies of the square plate with a central cutout.

BC	Methods	Modes					
		1	2	3	4	5	6
FFFF-F	Present	264.661	386.901	484.493	694.383	694.432	1235.285
	Experiment	257.680	375.950	469.310	674.490	678.260	1176.560
	Error(%)	2.638	2.830	3.134	2.865	2.329	4.754
CCCC-F	Present	732.448	1489.322	1489.796	2179.902	2642.341	2678.284
	Experiment	724.370	1448.740	1453.420	2102.870	2584.650	2608.280
	Error(%)	1.103	2.725	2.442	3.534	2.183	2.614

**Table 8:** The first six non-dimensional frequency parameters of the FGMs square plate with diverse cutout sizes.

Cutout Size Ratio *r/a*	Modes					
	1	2	3	4	5	6
0	25.5012	49.1018	49.1093	69.2248	81.9838	82.7225
0.025	25.4001	48.9005	48.9060	68.7938	81.4039	82.0926
0.05	25.3638	48.6206	48.6221	68.4080	80.8594	82.0073
0.075	25.4796	47.9396	47.9450	67.9465	80.2203	83.0599
0.1	25.8668	46.8228	46.8359	67.3965	79.4324	85.4772
0.125	26.6235	45.5138	45.5164	66.7080	78.3640	89.1572
0.15	27.8147	44.3487	44.3506	65.7601	76.8849	93.7810
0.175	29.5120	43.6500	43.6633	64.5325	75.1048	95.0332
0.2	31.8213	43.7553	43.7609	63.2576	73.5462	94.6269
0.225	34.8721	44.7776	44.7829	62.1695	72.6640	93.7299
0.25	38.8206	46.9072	46.9150	61.6343	73.0458	92.8681

**Table 9:** The first six non-dimensional frequency parameters of the FGMs square plate with diverse cutout positions.

Cutout Position *x_c_*	Modes					
	1	2	3	4	5	6
0.5	25.8668	46.8228	46.8359	67.3965	79.4324	85.4772
0.55	25.8376	46.8694	47.1622	67.3042	79.1040	83.9540
0.6	25.7714	47.0096	48.0869	67.1039	77.9703	82.3428
0.65	25.6586	47.2151	49.2026	66.8924	77.2401	82.1270
0.7	25.5036	47.4987	49.8348	66.8156	78.4654	82.8420
0.75	25.2945	47.8175	49.5779	66.9872	80.1950	84.1670
0.8	25.0313	48.1433	48.6649	67.4224	81.0591	83.9047

**Table 10:** The first six non-dimensional frequency parameters of the FGMs square plate with diverse cutout numbers.

Cutout Numbers	Modes					
	1	2	3	4	5	6
0	25.5012	49.1018	49.1093	69.2248	81.9838	82.7225
1	25.3638	48.6206	48.6221	68.4080	81.4594	82.0073
2	25.4322	48.4312	48.7497	68.2540	81.1932	81.7552
3	25.3848	48.0834	48.4720	67.6411	80.7516	80.8624
4	25.3126	47.8140	48.3909	67.1717	80.3960	80.4916

## Data Availability

The data presented in this study are available on request from the corresponding author.
